# Black Esophagus: A Rare Case of Acute Esophageal Necrosis Induced by Diabetic Ketoacidosis in a Young Adult Female

**DOI:** 10.1155/2018/7363406

**Published:** 2018-11-29

**Authors:** Zachary Field, Jacqueline Kropf, Meghan Lytle, Giselle Castaneira, Mario Madruga, S. J. Carlan

**Affiliations:** ^1^Department of Internal Medicine, Orlando Regional Healthcare, Orlando, FL, USA; ^2^Division of Academic Affairs and Research, Orlando Regional Healthcare, Orlando, FL, USA

## Abstract

**Background:**

Acute esophageal necrosis is an uncommon clinical disorder diagnosed on endoscopy as a black esophagus. It has a multifactorial etiology that probably represents a combination of poor nutritional status, gastric outlet obstruction, and ischemia secondary to hypoperfusion of the distal esophagus. It typically occurs in older males with comorbidities.

**Case:**

A 37-year-old woman presented with diabetic ketoacidosis and hematemesis. At esophagogastroduodenoscopy acute esophageal necrosis was diagnosed. The treatment included fluid and electrolyte management, insulin, and a proton pump inhibitor. She improved and left the hospital on day 3.

**Conclusion:**

Diabetic ketoacidosis can result in a profound osmotic diuresis, fluid loss, and hypoperfusion of the distal esophagus. This condition can then lead to ischemic injury and acute esophageal necrosis. Awareness of the possibility of its presence in young women with hematemesis and poorly controlled diabetes is important since early identification with esophagogastroduodenoscopy is necessary to prevent serious postnecrotic complications.

## 1. Introduction

Acute esophageal necrosis (AEN) is also known as black esophagus or Gurvits Syndrome. It was first described in the medical literature in 1990 by Goldenberg and colleagues but became a distinct clinical syndrome in 2007 by Gurvits [1, 2]. The incidence of the disorder is low and the diagnosis requires a high degree of clinical suspicion and visual inspection on esophagogastroduodenoscopy. It is virtually always associated with comorbidities including diabetes mellitus, malignancy, thromboembolic disorders, alcohol abuse, renal insufficiency, and cardiovascular disease [2] In fact, the degree of collective concurrent illness will, in large part, determine the prognosis. The mortality rate has been reported to be as high as 32%-36% which reflects the underlying illnesses [3, 4]. Treatment includes active management of the comorbidities, intravenous administration of a proton pump inhibitor, and possibly short-term parenteral nutrition. The main aim of treatment is to avoid extension of the injury and allow spontaneous healing. We present an unusual case of a young diabetic female with documented black esophagus.

## 2. Case Presentation

**Figure 1:**
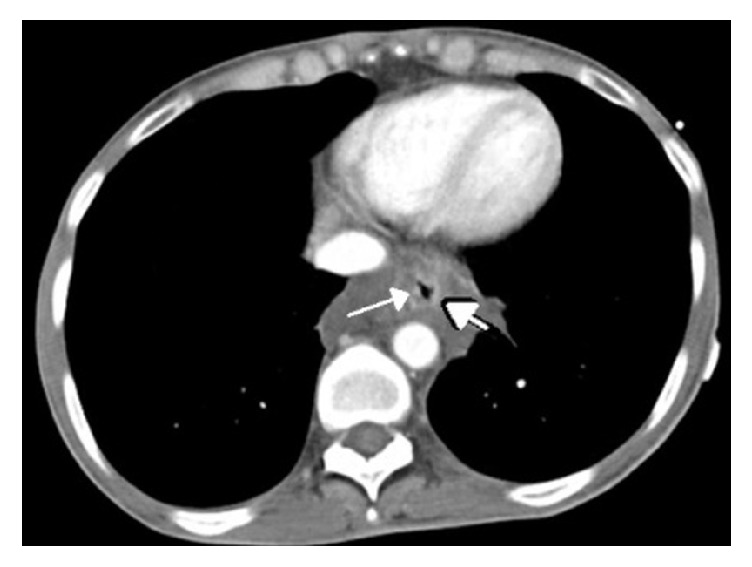
CT scan of the upper abdomen. Low-attenuation surrounding the distal esophagus with esophageal wall thickening (arrows).

**Figure 2:**
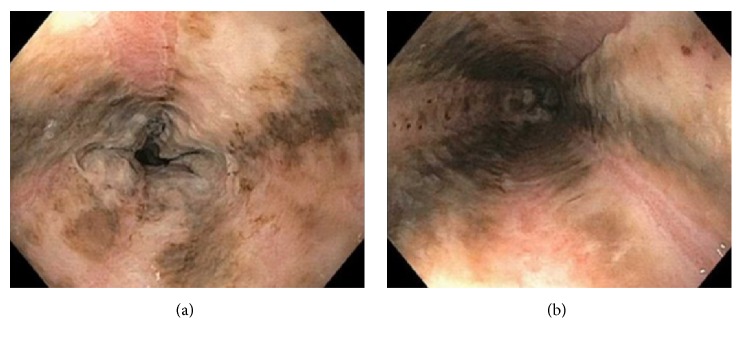
EGD image showing longitudinal ulceration and necrosis of the distal esophagus. Image (a) is slightly more distal and shows the significant mucosal necrosis and erosion. Image (b) shows the diffusely black appearance secondary to the ischemic insult.

A 37-year-old female with a past medical history significant for type I diabetes mellitus for > 20 years, Addison disease, and systemic lupus erythematosus presented to the emergency department with increasing abdominal pain over a six-day period. She also described poor oral intake and several episodes of nausea with blood-tinged emesis. She was treated 6 weeks earlier for* Clostridium difficile* colitis and completed a full course of antibiotics with resolution of symptoms; however over a week prior to the current admission her diarrhea had recurred. She denied any history of tobacco or alcohol use but did describe daily marijuana use. She was taking hydrocortisone 20 milligrams (mg) twice daily (bid) for Addison disease, insulin lispro 5 units (u) with meals, and insulin glargine 10 u at night. On admission her vital signs were temperature 97.1° F, blood pressure 130/102 mmHg, heart rate 140 beats/minute, and respiratory rate 26 breaths/minute. Her physical exam was only significant for abdominal guarding. The remainder of the physical exam was unremarkable. Metabolic panel demonstrated a blood glucose of 763 mg/dL, CO_2_ of 8 mmol/L, and *β*-hydroxybutyrate of 15.3 mmol/L with an anion gap of 36 mmol/L. Arterial blood gas (ABG) was significant for a metabolic acidosis with a pH of 7.11 and HCO_3_ of 4.1 mmol/L. Additional laboratory values were significant for urinary ketones of 80 mg/dL, urine glucose of ≥ 500 mg/dL, and a hemoglobin A1C of 15.3%. The findings were consistent with diabetic ketoacidosis (DKA) and she was started on an insulin drip with aggressive intravenous fluid resuscitation. Due to her significant abdominal pain, a computed tomography (CT) of the abdomen and pelvis was done which revealed extensive low-attenuation surrounding the distal esophagus with esophageal wall thickening (Figure 1). Gastroenterology was consulted in regard to the abnormal CT findings and the decision was made to perform an esophagogastroduodenoscopy (EGD) which revealed severe, diffuse esophageal ulcerations and necrosis (Figure 2). A diagnosis of necrotizing esophagitis was made. The patient was started on a pantoprazole drip, oral sucralfate, and a clear liquid diet. Her stool workup was negative and the diarrhea responded to antimotility agents. The anion gap closed, and blood sugars normalized. She improved clinically but left the hospital against medical advice on hospital day 3 before complete resolution of her symptoms.

## 3. Discussion

There are two unique elements to this case. First, AEN is a rare disorder, [5, 6] and, when found, is associated with a high mortality rate [5, 7]. The reported high mortality probably reflects two features. First, it is typically a disease of the elderly male who may have long-standing general malnutrition. Additionally, this group already is at risk for ischemic insults. The current theory of the pathophysiology of AEN is one of local distal esophageal ischemia that results from hypoperfusion secondary to hypovolemia from either third spacing or fluid loss. Since the lower esophagus is a watershed zone, the resultant compensatory shunting of blood away from the distal esophagus results in the ischemic injury that is evident on EGD [2]. If the ischemia lasts long enough the damage becomes progressive and complete healing is less likely. Consequently, a rapid diagnosis and medical remedy of the hypoperfusion by restoring normal fluid balance is critical to avoid esophageal perforation or stricture. The second element pertaining to the high mortality rate is that AEN is virtually always associated with comorbidities that may result in the hypoperfusion state. Intermittent esophageal ischemia and lower esophageal insults probably occur in this group of patients serially and frequently, but because the intravascular fluid balance is usually restored over the course of a few hours or days the mucosal damage is superficial and healing occurs [2]. Gastric outlet obstruction is not typically detected on EGD in AEN but is another factor that may contribute to local conditions that favor the development of AEN. Even when present in a transient form, gastric outlet obstruction and malnutrition are chronic conditions that can result in reflux, gastric stasis, gastric dilation, and an overall reduction in the mucosal defense mechanism against gastric contents. Our patient's 20-year history of type I diabetes may be a factor in gastric motility dysfunction.

The second unique element of this case is that this disorder is rare in the younger female population; thus it would be easy to overlook the possibility of AEN in that demographic. A timely diagnosis is fundamental to preventing long term consequences. Consequently, EGD should be considered early in the course of management even in the young female population if there is a suspicion based on history, exam, or imaging. Our case clearly demonstrated an abnormality in the distal esophagus on abdominal CT scan, but EGD is necessary for confirmation.

With the exception of cases resulting from caustic substance ingestion, AEN has not been reported in the pediatric group, even in type I diabetics, suggesting this disorder is the result of recurrent local ischemic and chemical insults to the distal esophagus.

Our patient presented with hematemesis, abdominal pain, and diarrhea. Hematemesis occurs in approximately 70% of cases. Other presenting features include vomiting, retrosternal chest discomfort, and dysphagia [1, 6]. Her diarrhea was likely a cofactor in her dehydrated state and may have been operative in the chronicity of her intravascular fluid balance. The role of her Addison disease and chronic hydrocortisone use could also be a factor with regard to fluid balance but she was not taking any mineralocorticoid. Diabetes and hydrocortisone use may, however, place her in an immunocompromised state and increase the risk of esophageal infection. Nonetheless, her EGD revealed the typical and expected findings of circumferential, diffusely necrotic distal esophageal mucosa terminating abruptly at the gastroesophageal junction and extending proximally [1, 3, 4]. A biopsy was not performed but the usual histological findings in AEN include necrotic material with lymphoplasma cell infiltration, polymorphonuclear neutrophils, and eosinophils [2, 5]. Endoscopically biopsied tissue can be sent for viral and bacterial culture and fungal staining, especially in immunocompromised individuals, but her EGD was not consistent with an infectious process and clinically she was improving.

The correlation between hyperglycemia and AEN has been well described in the literature. Up to 90% of patients with AEN are found to be at least moderately hyperglycemic at the time of presentation [6]. Though hyperglycemia is common in AEN, few cases of AEN in the setting of DKA have been reported. Few hypotheses exist regarding the link between DKA and AEN, but hypoperfusion and ischemic insults are likely operative. Our case has a unique component to her type I diabetes that may be a risk factor for a lower threshold for esophageal ischemia. She reportedly had poor glycemic control for over 20 years which would be consistent with intrinsic vascular disease further increasing the risk of both gastric dysmotility and ischemia. In fact, her HgBA1C on admission was 15.3. The distal esophagus has an intrinsic barrier system that protects itself from reflux of acidic gastric contents [2]. This barrier system can be damaged in the setting of uncontrolled diabetes, which often leads to delayed esophageal peristalsis and gastric emptying, further predisposing the esophagus to cell damage and necrosis [2, 7].

As a rare, understudied clinical entity, no standardized therapies for acute esophageal necrosis currently exist. Treatment should be directed at correcting the underlying medical condition with hemodynamic resuscitation and glycemic control [2, 4, 5]. Additionally, maintenance of nil-per-os status and acid-suppression therapy with an intravenous, high-dose proton pump inhibitor and sucralfate are recommended [2, 4, 5]. Overall, the prognosis of AEN is poor which is usually related to the underlying condition rather than AEN itself [2, 6]. Long-term complications of the AEN include esophageal stenosis, stricture formation, and esophageal perforation [2, 3]. Stricture formation occurs with an incidence of 25% and may require endoscopic balloon dilation [2, 4, 6, 7]. Though esophageal perforation occurs in less than 7% of patients, perforation can be fatal if not recognized and treated immediately with surgical intervention [5, 6]. Although it remains a rare cause of upper gastrointestinal bleeding, AEN should be considered as a potential diagnosis in young adult females presenting with DKA or another hyperglycemic state with associated comorbidities. Ruling out other etiologies of AEN is essential as treating the underlying cause is part of the mainstay of therapy. With a high mortality rate and significant complications if recognition and treatment are delayed, early endoscopic utilization is critical to improving patient outcomes.
